# Transcriptomic signatures responding to PKM2 activator TEPP-46 in the hyperglycemic human renal proximal epithelial tubular cells

**DOI:** 10.3389/fendo.2022.965379

**Published:** 2022-08-31

**Authors:** Zhimin Wang, Jiating Yu, Dan Hao, Xin Liu, Xiao Wang

**Affiliations:** ^1^ Division of Endocrinology and Metabolic Diseases, The First Affiliated Hospital of Zhengzhou University, Zhengzhou, China; ^2^ Division of Pediatric Surgery, The First Affiliated Hospital of Zhengzhou University, Zhengzhou, China; ^3^ Shijiazhuang Zhongnongtongchuang (ZNTC) Biotechnology Co., Ltd., Shijiazhuang, China; ^4^ Department of Biology, University of Copenhagen, Copenhagen, Denmark; ^5^ Division of Clinical Laboratory, Key Clinical Laboratory of Henan Province, The First Affiliated Hospital of Zhengzhou University, Zhengzhou, China; ^6^ Konge Larsen ApS, Kongens Lyngby, Denmark; ^7^ College of Animal Science and Technology, Qingdao Agricultural University, Qingdao, China

**Keywords:** transcriptomic analysis, human renal proximal epithelial tubular cells, high D-glucose, TEPP-46, diabetic kidney disease

## Abstract

Pyruvate kinase M2 (PKM2), as the terminal and last rate-limiting enzyme of the glycolytic pathway, is an ideal enzyme for regulating metabolic phenotype. PKM2 tetramer activation has shown a protective role against diabetic kidney disease (DKD). However, the molecular mechanisms involved in diabetic tubular have not been investigated so far. In this study, we performed transcriptome gene expression profiling in human renal proximal tubular epithelial cell line (HK-2 cells) treated with 25 mM high D-glucose (HG) for 7 days before the addition of 10 μM TEPP-46, an activator of PKM2 tetramerization, for a further 1 day in the presence of HG. Afterwards, we analyzed the differentially expressed (DE) genes and investigated gene relationships based on weighted gene co-expression network analysis. The results showed that 2,902 DE genes were identified (adjusted *P*-value ≤ 0.05), where 2,509 DE genes (86.46%) were co-expressed in the key module. Four extremely downregulated DE genes (*HSPA8*, *HSPA2*, *HSPA1B*, and *ARRB1*) and three extremely upregulated DE genes (*GADD45A*, *IGFBP3*, and *SIAH1*) enriched in the downregulated endocytosis (hsa04144) and upregulated p53 signaling pathway (hsa04115), respectively, were validated by qRT-PCR experiments. The qRT-PCR results showed that the relative expression levels of *HSPA8* [adjusted *P*-value = 4.45 × 10^-34^ and log_2_(FC) = -1.12], *HSPA2* [adjusted *P*-value = 6.09 × 10^-14^ and log_2_(FC) = -1.27], *HSPA1B* [adjusted *P*-value = 1.14 × 10^-11^ and log_2_(FC) = -1.02], and *ARRB1* [adjusted *P*-value = 2.60 × 10^-5^ and log_2_(FC) = -1.13] were significantly different (*P*-value < 0.05) from the case group to the control group. Furthermore, the interactions and predicted microRNAs of the key genes (*HSPA8*, *HSPA2*, *HSPA1B*, and *ARRB1*) were visualized in networks. This study identified the key candidate transcriptomic biomarkers and biological pathways in hyperglycemic HK-2 cells responding to the PKM2 activator TEPP-46 that can highlight a possibility of PKM2 tetramerization reshaping the interplay among endocytic trafficking through the versatile networks of Hsp70s and rewiring the crosstalk between EGFR signal transduction circuits and metabolic stress to promote resilience, which will be valuable for further research on PKM2 in DKD.

## Introduction

Diabetic kidney disease (DKD) is continuing to impose the highest burden and the strongest correlation with mortality due to the increasing diabetes incidence globally (1). The presentation of DKD in the clinic shows an extreme phenotypic heterogeneity (2). Patients with diabetes will not always develop clinically evident DKD even in the setting of poor glycemic control. This heterogeneous clinical cohorts derived the impetus to uncover endogenous protective factors against the development of DKD (3). The ratio between the tetramer and dimer structure of pyruvate kinase M2 (PKM2) mediates cellular glycolytic reshuffling and metabolic phenotype through its precise allosteric regulation, acting as a keystone in cellular glycolytic remodeling (4, 5). Tetrameric PKM2 has shown a protective role against the progression of DKD and chronic kidney disease (CKD) in type 1 and type 2 diabetes from clinical individuals, partly by rewiring the plethora of carbon compounds to catabolism, reducing toxic glucose metabolites, and thereby circumventing energy consumption and redox stress from synthesis (3, 6). The small-molecule allosteric agent TEPP-46 could transfer the dimeric PKM2 from a loose state (T-state) to a stable and compact state (R-state) to form a tetramer by binding to a pocket of the PKM2 subunit interface (5, 7). PKM2 tetramerization by TEPP-46 possesses high PK enzyme activity and blocks its nuclear translocation (5). TEPP-46 could restore hyperglycemia-induced glomerular and tubular metabolic phenotypes, further inhibiting fibrosis progression in DKD (3, 8).

Recently, the clinical relevance of decreased proximal reabsorption and hyperfiltration by sodium glucose cotransporter 2 inhibitors (SGLT2i) to improve the renal outcomes in patients with diabetes revolutionized the treatment of DKD and CKD, further establishing the core status of the renal proximal tubular center (9). Proximal tubular epithelial cells (PTECs) are metabolically active to impose a high energy demand for its reabsorption and transport processes. Due to its physiologic role, PTECs are susceptible to nutritional stress such as in the diabetic context. Increased reabsorption of glucose and sodium in PTECs under chronic hyperglycemic stress triggers renal hyperfiltration and exacerbates hypoxia, playing a crucial role in preserving the diabetic state and renal injury (10, 11). Compared with other metabolically active cells such as cardiomyocytes or skeletal muscle cells, PTECs show a relatively low metabolic flexibility toward glycolysis and mainly rely on fatty acids as energy source (12). Glycolytic adaptation or maladaptation of PTECs couples metabolic resilience to cell fate and the process of DKD, which remains poorly understood.

In our study, we explored the transcriptomic changes and mechanisms in response to PKM2 activator TEPP-46 in the hyperglycemic human renal proximal epithelial tubular cell line (HK-2 cells) *in vitro* to reveal the pathophysiological networks involved in the glycolytic reprogramming of hyperglycemic PTECs and thus to update and reshape the prevention strategy for DKD. A total of six independent replicates of HK-2 cells with three replicates in the case/control groups were used. We conducted a genome-wide transcriptome study in HK-2 cells cultured with high D-glucose (HG) for 7 days with (case group) or without (control group) the addition of TEPP-46 for another 1 day in the presence of HG to reveal differentially expressed (DE) genes; meanwhile, the gene co-expression network relationships are constructed by the weighted gene co-expression network analysis (WGCNA) tool (13) because the co-expressed genes with similar expression patterns across samples are controlled by the same transcriptional regulatory programs (14, 15). A DE gene-related biological enrichment analysis was subsequently performed to identify the potential functions in the major module of co-expression networks to reveal the candidate biomarkers to provide new mechanistic opportunities to remodel the cell resilience of PTECs.

## Materials and methods

### Cell culture and transcriptomic sequencing

Cultured HK-2 cells were obtained from Beijing Beina Chuanglian Biotechnology Research Institute, where they were cultured in Dulbecco’s modified eagle medium with low glucose (Sigma-Aldrich Inc., St. Louis, MO, USA) supplemented with 10% fetal bovine serum (Sigma-Aldrich, St. Louis, MO, USA), 10 units/ml penicillin, and 10 mg/ml streptomycin and maintained in a continuous culture at 37°C in a humidified atmosphere (5% CO_2_) in an incubator. The growth medium was shifted every 2 or 3 days, and the cells were sub-cultured until further measurements at 80% colony confluency. The obtained HK-2 cells were exposed to 25 mM HG medium as the hyperglycemic condition for 7 days. Thereafter, three replicates of cells exposed to HG were separately treated with (case group) or without (control group) 10 μM TEPP-46 for 1 day. We extracted 2 μg RNA per cell replicate sample for the RNA sample preparation, generated the sequencing libraries using NEBNext Library Prep Kit (NEB, USA) following the manufacturer’s recommendations, and sequenced these on the Illumina Hiseq X Ten platform to generate the 150-bp paired-end reads step by step.

### Quality control, alignment, and differentially expressed analysis

Quality control of raw data was done based on three criteria: the contaminated reads for adapters (> 5 bp adapter sequences), low quality reads (Phred quality value ≤19 more than 15%), and reads with Ns (Ns >5%). The filtered clean reads were then aligned to the human reference genome GRCh38.p13 (Genome Reference Consortium Human Build 38) using HISAT2 software (version 2.1.0) (16). Afterwards, the fragments per kilobase million mapped reads (FPKM) (**Supplementary File S1**) (17, 18) were calculated based on the read counts derived for each gene using HTSeq software (version 0.6.0) (19). We used R package DESeq2 (version 1.30.0) for DE gene analysis (20–22) and then calculated the *P*-value by Wald’s test and corrected the multiple testing by the Benjamini–Hochberg method to achieve the adjusted *P*-value. In this study, we considered the analyzed genes of adjusted *P*-value ≤0.05 as the DE genes and those log_2_(FC) >0 and log_2_(FC) <0 as upregulated and downregulated genes, respectively.

### Major module of the co-expression network and their associations with TEPP-46 treatment

We constructed the gene co-expression network for the similarity measurement between the gene expression profiles by Pearson correlation coefficients of the matrix using R package WGCNA (13). A total of 21,067 genes were filtered out of 29,483 genes that were used for DE analysis based on the median absolute deviation ≥0.01 in this study. The β power parameter (soft threshold) was equal to 5 when the *R* ^2^ of the free-scale topology was equal to 0.8 (**Supplementary Figure S1**). The modules whose eigengenes are correlated above 0.75 were merged, as the cut height for the modules was set to merge at 0.25. Module association between the module eigengenes and the TEPP-46 treatment status (*i*.*e*., 0, 0, 0, 1, 1, 1 for the control and case groups, respectively) was calculated for the relevant module identifications. The module significance (MS) was calculated to evaluate the correlations, *i*.*e*., the module with the highest MS score belongs to the key module (13), where top MS genes in the association analysis (*P*-value < 0.1) were assigned for functional enrichment analysis.

### Biological pathway enrichment analysis of differentially expressed genes in the key module

The biological Kyoto Encyclopedia of Genes and Genomes (KEGG) pathway analysis was investigated for the important enrichments in the associations between the top MS genes and the gene-related biological functions. R package *clusterProfiler* (version 3.6) (23) was applied to test the statistical enrichments of KEGG pathways with DE genes in the key modules with high MS scores.

### Protein–protein interactive analysis and predicted microRNAs based on the candidate differentially expressed genes

Based on the STRING database (https://string-db.org) (24), the interaction scores were calculated and the protein–protein interactions (PPIs) were predicted for the candidate DE genes. The PPI networks were built according to the known interactions of human species and visualized by the Cytoscape software (version 3.5.1) (25). The target microRNA (conserved sites) predictions of the candidate DE genes were performed using TargetScan software (version 7.0) that provided the ranks based on the predicted regression according to the cumulative weighted context++score (CWCS) (26).

### Experimental validations

Qualitative reverse transcription polymerase chain reaction (qRT-PCR) was used to validate candidate DE genes using the re-cultured and re-treated cells. The method of 2−^ΔΔCt^ was used to calculate the relative gene expression levels. Glyceraldehyde-3-phosphate dehydrogenase (*GAPDH*) was chosen as the internal gene for qRT-PCR experiments, and 2× SYBR Green qPCR Master Mix (High ROX) (Servicebio, Wuhan, China) was used to perform the qRT-PCR experiments. All the primers for the qRT-PCR experiments are shown in **Supplementary Table S1**.

## Results

### Statistics of quality control and alignment

After quality control, the average of 45,781,340 (96.75%) clean reads were filtered from 47,317,956 raw reads by removing 1,160,096 (2.45%) adapter polluted reads, 251,479 (0.53%) low-quality reads, and 125,041 (0.27%) ploy-N reads. The 7,097,693,400 raw bases were also filtered to 6,867,201,000 clean bases, while the percentage of the Q30 bases increased to 93.13 from 92.84% (**Table 1**). Afterwards, 44,472,637 of them were uniquely mapped to the human reference genome with a mapping rate of 97.14%, where 18,261,167 (41.06%) and 902,185 (2.03%) uniquely mapped reads were located in the exon and intron regions, respectively (**Table 1**).

**Table 1:** Statistics of quality control and alignment to human reference genome (GRCh38.p13).

Sample	Raw read number	Raw base number (Q30 base rate %)	Adapter polluted read number (%)	Low-quality read number (%)	Ploy-N read number (%)	Clean read number (%)	Clean base number (Q30 base rate %)	Mapped clean read number to reference genome (%)	Mapped clean read number (%) in exons of reference genes	Mapped clean read number (%) in introns of reference genes
HG1	48,909,654	7,336,448,100 (92.98)	732,722 (1.50)	219,180 (0.45)	70,820 (0.14)	47,886,932 (97.91)	7,183,039,800 (93.20)	46,597,334 (97.31)	19,291,203 (41.40)	806,613 (1.73)
HG2	47,359,384	7,103,907,600 (92.80)	675,864 (1.43)	337,124 (0.71)	83,178 (0.18)	46,263,218 (97.69)	6,939,482,700 (93.13)	44,866,400 (96.98)	18,748,424 (41.79)	818,605 (1.82)
HG3	47,542,838	7,131,425,700 (92.84)	2,126,872 (4.47)	192,338 (0.41)	261,400 (0.55)	44,962,228 (94.57)	6,744,334,200 (93.17)	43,678,133 (97.14)	18,195,761 (41.66)	781,807 (1.79)
HGTEPP1	47,027,902	7,054,185,300 (93.01)	2,045,470 (4.35)	171,772 (0.36)	173,294 (0.37)	44,637,366 (94.92)	6,695,604,900 (93.26)	43,421,950 (97.28)	18,183,221 (41.88)	661,783 (1.52)
HGTEPP2	46,777,790	7,016,668,500 (93.24)	633,516 (1.35)	311,876 (0.67)	88,754 (0.19)	45,743,644 (97.79)	6,861,546,600 (93.55)	44,481,632 (97.24)	17,597,949 (39.56)	1,224,360 (2.75)
HGTEPP3	46,290,168	6,943,525,200 (92.17)	746,130 (1.61)	276,586 (0.60)	72,800 (0.16)	45,194,652 (97.63)	6,779,197,800 (92.44)	43,790,373 (96.89)	17,550,446 (40.08)	1,119,942 (2.56)
Mean	47,317,956	7,097,693,400 (92.84)	1,160,096 (2.45)	251,479 (0.53)	125,041 (0.27)	45,781,340 (96.75)	6,867,201,000 (93.13)	44,472,637 (97.14)	18,261,167 (41.06)	902,185 (2.03)
SD	818,337	122,750,502 (0.33)	656,293 (1.39)	61,247 (0.13)	70,309 (0.15)	1,079,426 (1.43)	161,913,969 (0.34)	1,070,057 (0.16)	612,880 (0.90)	199,841 (0.46)

% indicates the percentage. HGTEPP1, HGTEPP2, and HGTEPP3 indicate the case groups that were treated with 10 μM TEPP-46 in 25 mM HG medium. HG1, HG2, and HG3 indicate the control groups that were treated without TEPP-46 in 25 mM HG medium.

### Identified differentially expressed genes

In this study, we identified 2,902 DE genes (adjusted *P*-value ≤ 0.05) including 1,413 downregulated DE genes and 1,489 upregulated DE genes. All the details of the 2,902 DE genes with chromosome positions, gene names, gene descriptions, read counts for each gene along with different samples, log_2_(FC) values, *P*-values, adjusted *P*-values, and regulation status are listed in the **Supplementary File S2**, where the top 10 downregulated DE genes and upregulated DE genes are shown in **Table 2**. We found that the most downregulated DE gene was *HSPA8* [log_2_(FC) = -1.12], while the most upregulated DE gene was *PPP1R15A* [log_2_(FC) = 1.15]. The log_2_(FC) values varied from -1.36 to 1.64 among the top 10 downregulated DE genes and upregulated DE genes (**Table 2**). Moreover, 143 out of 2,902 DE genes were recognized as extremely filtered according to the criterion of |log_2_(FC)| ≥ 1, where 87 and 56 of them were the extremely downregulated and upregulated DE genes, respectively (**Supplementary File S2**).

**Table 2:** Top 10 downregulated and upregulated differentially expressed genes.

Ensembl gene ID	Gene name	Full description	log_2_(FC)	Adjusted *P*-value	Regulation status
ENSG00000109971	*HSPA8*	Heat shock protein family A (Hsp70) member 8	-1.12	4.45 × 10^-34^	Down
ENSG00000134369	*NAV1*	Neuron navigator 1	-1.31	1.83 × 10^-20^	Down
ENSG00000168497	*SDPR*	Sporulation delaying system autorepressor	-1.23	2.38 × 10^-18^	Down
ENSG00000143375	*CGN*	Cingulin	-1.13	3.52 × 10^-15^	Down
ENSG00000126803	*HSPA2*	Heat shock protein family A (Hsp70) member 2	-1.27	6.09 × 10^-14^	Down
ENSG00000135074	*ADAM19*	ADAM metallopeptidase domain 19	-1.05	5.97 × 10^-13^	Down
ENSG00000013588	*GPRC5A*	G protein-coupled receptor class C group 5 member A	-0.76	1.86 × 10^-12^	Down
ENSG00000196535	*MYO18A*	Myosin XVIIIA	-1.04	5.57 × 10^-12^	Down
ENSG00000142798	*HSPG2*	Heparan sulfate proteoglycan 2	-1.36	1.14 × 10^-11^	Down
ENSG00000204388	*HSPA1B*	Heat shock protein family A (Hsp70) member 1B	-1.02	1.14 × 10^-11^	Down
ENSG00000087074	*PPP1R15A*	Protein phosphatase 1 regulatory subunit 15A	1.51	5.12 × 10^-43^	Up
ENSG00000255717	*SNHG1*	Small nucleolar RNA host gene 1	1.48	6.14 × 10^-37^	Up
ENSG00000169715	*MT1E*	Metallothionein 1E	1.45	9.03 × 10^-37^	Up
ENSG00000140961	*OSGIN1*	Oxidative stress induced growth inhibitor 1	1.41	4.76 × 10^-30^	Up
ENSG00000081041	*CXCL2*	C-X-C motif chemokine ligand 2	1.08	5.00 × 10^-28^	Up
ENSG00000170385	*SLC30A1*	Solute carrier family 30 member 1	1.36	8.76 × 10^-28^	Up
ENSG00000123689	*G0S2*	G0/G1 switch 2	1.31	1.97 × 10^-26^	Up
ENSG00000175197	*DDIT3*	DNA damage inducible transcript 3	1.64	9.45 × 10^-26^	Up
ENSG00000101255	*TRIB3*	Tribbles pseudokinase 3	1.05	5.26 × 10^-24^	Up
ENSG00000116717	*GADD45A*	Growth arrest and DNA damage inducible alpha	1.06	3.44 × 10^-19^	Up

**Figure 1:**
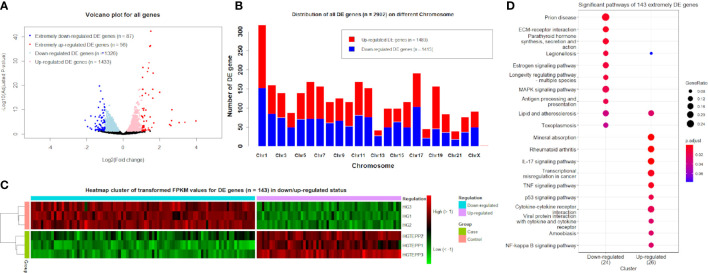
Identified differentially expressed (DE) genes. **(A)** Volcano plots for all genes based on log_2_(FC) values and adjusted *P*-values. Genes with thresholds of adjusted *P*-value ≤0.05 are identified as DE genes, while genes with thresholds of adjusted *P*-value ≤0.05 and |log_2_(FC)| ≥1 are identified as extremely DE genes. **(B)** Distribution of DE genes (*n* = 2,902) on different chromosomes. **(C)** Heat map cluster for DE genes (*n* = 143) in downregulated and upregulated status. The colors from green to red indicate the gene expression levels from low to high after the transform of log_10_(FPKM + 1). HGTEPP1, HGTEPP2, and HGTEPP3 indicate the three replicate samples in the case groups that were treated with 10 μM TEPP-46 in 25 mM HG medium, while the rest of the three samples are in the control groups that were treated without TEPP-46 in 25 mM HG medium. **(D)** Significant pathways for extremely DE genes (*n* = 143) under the downregulated and upregulated categories.

The obvious division between the upregulated DE genes and downregulated DE genes based on log_2_(FC) values can be observed, where the range of log_2_(FC) values reached from -2 to 4 for the downregulated and upregulated DE genes (**Figure 1A**). The downregulated and upregulated DE genes were evenly located on different chromosomes, whereas chromosome 19 appeared to have more upregulated DE genes (*n* = 111) than downregulated genes (*n* = 46) (**Figure 1B**). On chromosome 13, the upregulated DE genes (*n* = 14) and downregulated genes (*n* = 28) are unevenly distributed as well. With the additional filtering of |log_2_(FC)| ≥1, the transformed FPKM values of 143 extremely filtered DE genes were clustered into case/control groups separately. Meanwhile, in the downregulated status, the gene expressions of the control group showed apparently higher levels than the case group and *vice versa* (**Figure 1C**). The top three significant pathways for the 143 extremely DE genes were prion disease (hsa05020, adjusted *P*-value = 5.65 × 10^-3^), extracellular matrix–receptor interaction (hsa04512, adjusted *P*-value = 5.65 × 10^-3^), and parathyroid hormone synthesis, secretion, and action (hsa04928, adjusted *P*-value = 7.74 × 10^-3^) in the downregulated category (**Figure 1D**). In the upregulated category, they were mineral absorption (hsa04978, adjusted *P*-value = 7.20 × 10^-5^), rheumatoid arthritis (hsa05323, adjusted *P*-value = 2.24 × 10^-4^), and IL-17 signaling pathway (hsa04657, adjusted *P*-value = 2.24 × 10^-4^) (**Figure 1D**).

### The key module of the co-expressions for all genes

**Figure 2:**
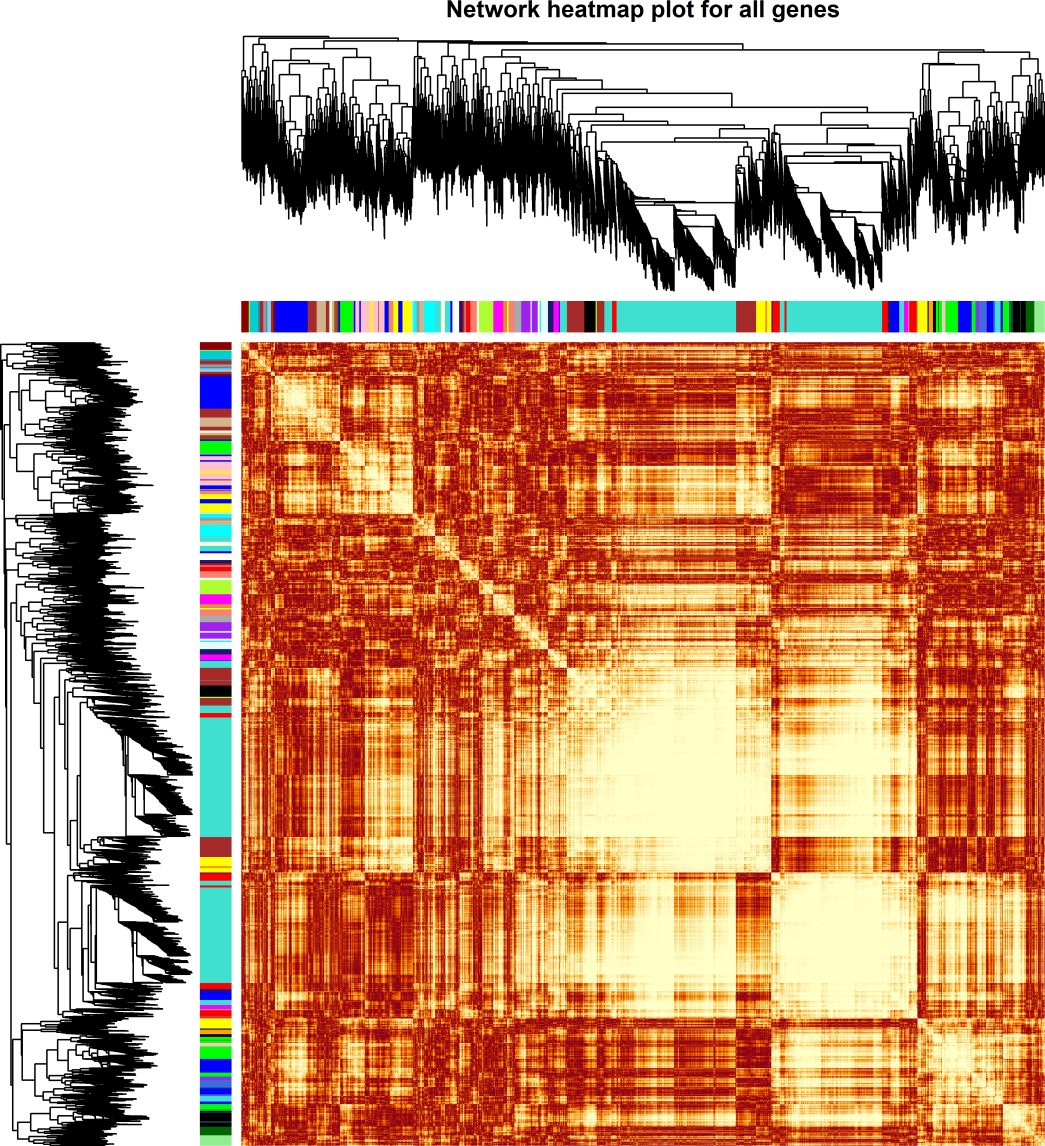
Weighted differentially expressed (DE) gene network heat map with assigned modules. The gene dendrogram and module assignment are shown along the left side and at the top, where the genes were clustered in the dendrogram with dissimilarity based on topological overlap and the axe colors indicate the different modules. The color intensity inside the heat map represents the overlap degree, where the lighter color represents a low overlap and the darker red color represents a higher overlap.

**Figure 3:**
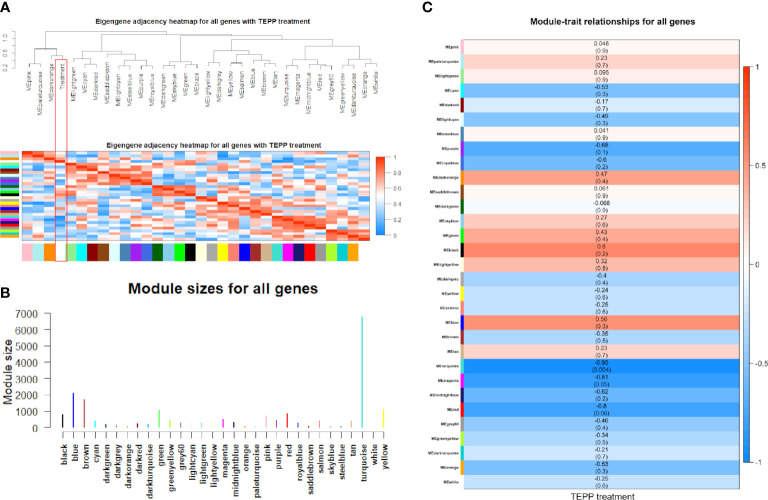
Co-expressions of differentially expressed (DE) genes. **(A)** Eigengene dendrogram and adjacency heat map with TEPP-46 treatment (red rectangle). **(B)** Module sizes (number of genes) in different colors. **(C)** Module–TEPP relationship heatmap. Each row indicates module eigengenes with the correlation coefficients (*P*-values in brackets), where red and blue colors represent positive and negative correlations, respectively.

The weighted DE gene network of the co-expression interactions showed a high level of overlap densities among 31 modules that was visualized in the topological overlap matrix clusters with the assigned module colors using the average linkage hierarchical clustering algorithm (**Figure 2**). The case group with TEPP-46 treatment was clustered with the dark orange module into a group, which was far from the key clustered turquoise, blue, and brown modules (**Figure 3A**). The 31 modules included the genes with similar co-expressions, where 6,770 genes were grouped into turquoise module as the key module, followed by 2,112 genes into blue module and 1,717 genes into brown module (**Figure 3B**). The module–TEPP relationship results showed that the TEPP-46 treatment status had a highly negative correlation with the turquoise module (-0.95, *P*-value = 0.004) and the magenta module (-0.81, *P*-value = 0.05) (**Figure 3C**).

### Significant pathway enrichment in the key module

Out of 2,902 DE genes, we found that 2,509 of them (86.46%) were co-expressed in the turquoise module, while the blue and brown modules only had 141 (4.86%) and three DE (0.1%) genes, respectively (**Table 3**). Afterwards, we used 1,354 (46.66%) downregulated DE genes and 1,155 (39.8%) upregulated DE genes in the turquoise module and 11 (0.38%) downregulated DE genes and 130 (4.48%) upregulated DE genes in the blue module to perform the pathway enrichment.

**Table 3:** Number of all differentially expressed (DE) genes in different co-expressed modules.

Co-expression module	Number of DE genes (%)	Number of downregulated DE genes (%)	Number of upregulated DE genes (%)
Turquoise	2,509 (86.46)	1,354 (46.66)	1,155 (39.80)
Blue	141 (4.86)	11 (0.38)	130 (4.48)
Brown	3 (0.10)	0 (0)	3 (0.10)
Yellow	2 (0.07)	0 (0)	2 (0.07)
Green	52 (1.79)	0 (0)	52 (1.79)
Red	81 (2.79)	31 (1.07)	50 (1.72)
Black	50 (1.72)	0 (0)	50 (1.72)
Pink	2 (0.07)	0 (0)	2 (0.07)
Magenta	36 (1.24)	8 (0.28)	28 (0.96)
Purple	5 (0.17)	4 (0.14)	1 (0.03)
Midnight blue	5 (0.17)	4 (0.14)	1 (0.03)
Light cyan	3 (0.10)	0 (0)	3 (0.10)
Royal blue	13 (0.45)	1 (0.03)	12 (0.41)
Total	2,902 (100.00)	1,413 (48.69)	1,489 (51.31)

**Figure 4:**
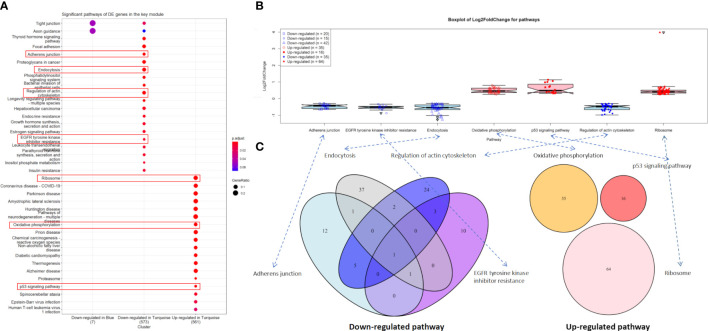
Significant pathways. **(A)** Significant pathways for differentially expressed (DE) genes in the turquoise (*n* = 2,509) and blue modules (*n* = 141) under the downregulated and upregulated categories. **(B)** Boxplot of log_2_(FC) values for endocytosis and p53 signaling pathway. **(C)** Venn diagram for the enriched DE genes of four downregulated and three upregulated pathways.

In the downregulated category, the top three significant pathways in the turquoise module were thyroid hormone signaling pathway (hsa04919, adjusted *P*-value = 3.67 × 10^-6^) with 28 DE genes, focal adhesion (hsa04510, adjusted *P*-value = 4.61 × 10^-6^) with 37 DE genes, and adherens junction (hsa04520, adjusted *P*-value = 4.61 × 10^-6^) with 20 DE genes. In the upregulated category, ribosome (hsa03010, adjusted *P*-value = 1.90 × 10^-31^) with 64 DE genes, coronavirus disease—COVID-19 (hsa05171, adjusted *P*-value = 4.33 × 10^-15^) with 56 DE genes, and Parkinson’s disease (hsa05012, adjusted *P*-value = 2.72 × 10^-14^) with 59 DE genes were the three most significant pathways in the turquoise module. However, no significant pathway was found in the blue module (**Figure 4A**).

Notably, 42 downregulated DE genes were enriched in endocytosis (hsa04144, adjusted *P*-value = 6.06 × 10^-6^). Four of these genes, *i*.*e*., *HSPA8* [log_2_(FC) = -1.12, adjusted *P*-value = 4.45 × 10^-34^], *HSPA2* [log_2_(FC) = -1.27, adjusted *P*-value = 6.09 × 10^-14^], *HSPA1B* [log_2_(FC) = -1.02, adjusted *P*-value = 1.14 × 10^-11^], and *ARRB1* [log_2_(FC) = -1.13, adjusted *P*-value = 2.60 × 10^-5^] were extremely downregulated DE genes (**Figure 4B** and **Supplementary File S2**). Similarly, 16 upregulated DE genes were enriched in the p53 signaling pathway (hsa04115, adjusted *P*-value = 5.90 × 10^-4^), including *AIFM2*, *BBC3*, *BCL2L1*, *CCNB2*, *CDK2*, *CDKN1A*, *CHEK1*, *COP1*, *DDB2*, *GADD45A*, *GADD45B*, *IGFBP3*, *PERP*, *SESN2*, *SIAH1*, and *TP53I3*, where *GADD45A* [log_2_(FC) = 1.06, adjusted *P*-value = 3.62 × 10^-22^], *IGFBP3* [log_2_(FC) = 1.00, adjusted *P*-value = 3.72 × 10^-8^], and *SIAH1* [log_2_(FC) = 1.13, adjusted *P*-value = 1.14 × 10^-7^] were the extremely upregulated DE genes (**Figure 4B** and **Supplementary File S2**).

For two other downregulated pathways, adherens junction (hsa04520, adjusted *P*-value = 4.89 × 10^-6^) and regulation of actin cytoskeleton (hsa04810, adjusted *P*-value = 1.18 × 10^-4^) contained 20 and 35 enriched DE genes, respectively (**Figure 4B**), where six of these genes were overlapped in both pathways—these were *ACTB*, *ACTG1*, *ACTN4*, *EGFR*, *IQGAP1*, and *VCL* (**Figure 4C**). We found that three downregulated pathways (adherens junction, endocytosis, and EGFR tyrosine kinase inhibitor resistance) contain *IGF1R* [log_2_(FC) = -0.61, adjusted *P*-value = 3.96 × 10^-5^]. Most importantly, *EGFR* [log_2_(FC) = -0.34, adjusted *P*-value = 2.91 × 10^-2^] was involved in all four downregulated pathways (adherens junction, endocytosis, EGFR tyrosine kinase inhibitor resistance, and regulation of actin cytoskeleton). Unfortunately, no overlapped DE gene was found in the three upregulated pathways (**Figure 4C**).

### Protein–protein interaction networks

**Figure 5:**
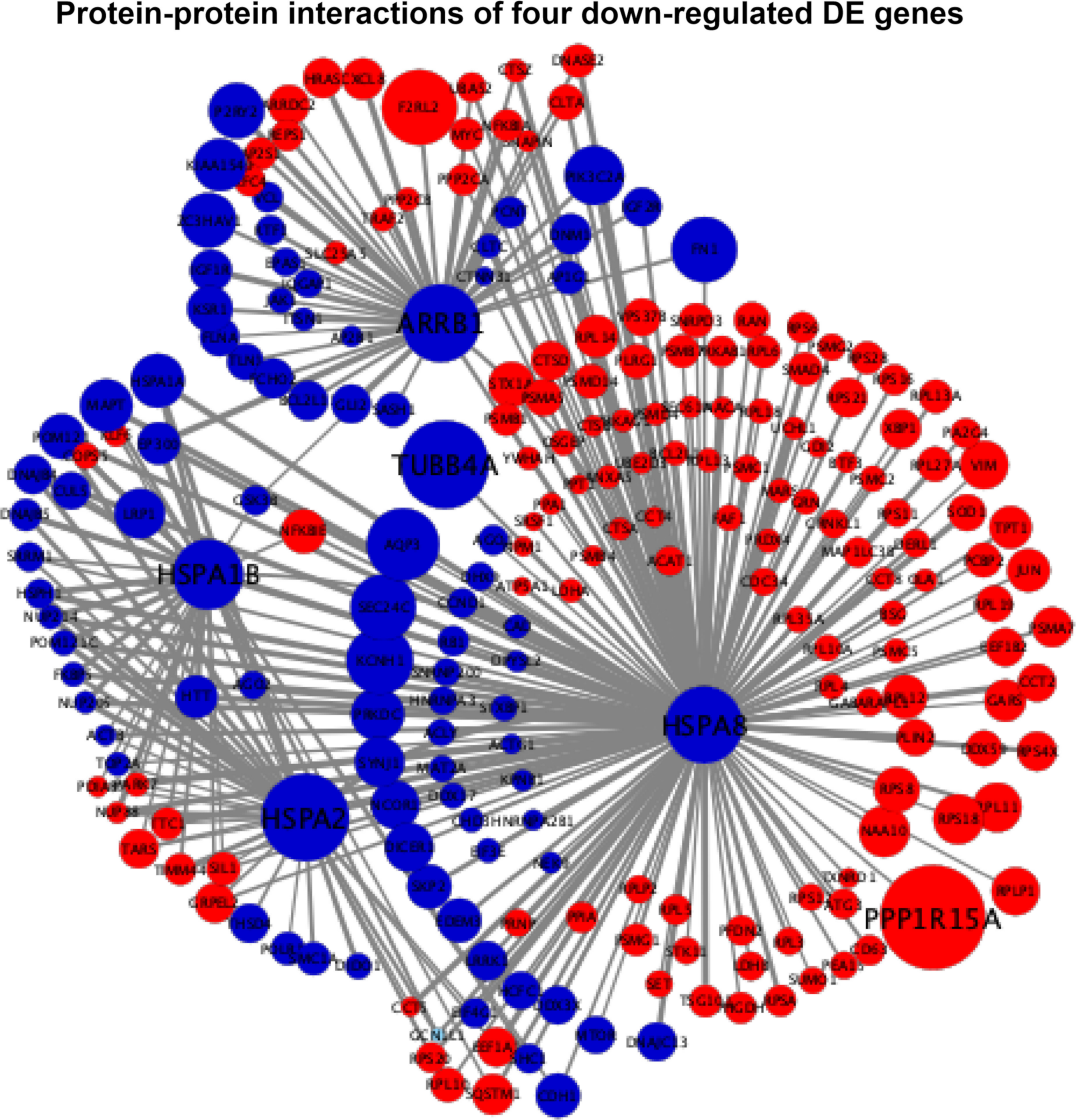
Protein–protein interaction (PPI) networks of four extremely downregulated differentially expressed genes (*HSPA8*, *HSPA2*, *HSPA1B*, and *ARRB1*). The circles indicate the nodes (genes) with the edges that are solid lines indicating the interaction scores of PPIs. The sizes of circles indicate the values of |log_2_(FC)|, while the red and blue colors indicate the upregulated and downregulated statuses, respectively.

**Figure 6:**
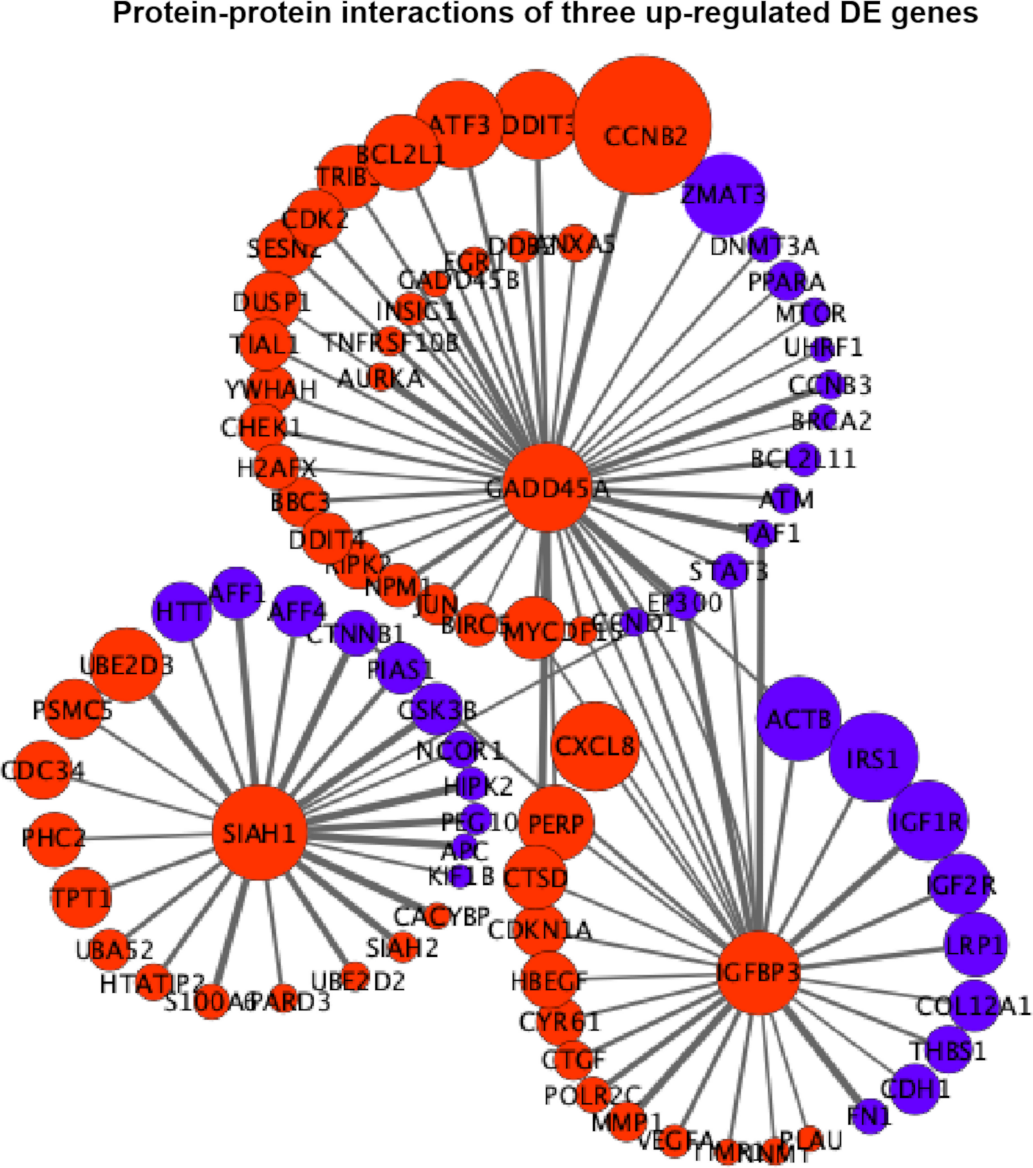
Protein–protein interaction (PPI) networks of three extremely upregulated differentially expressed (DE) genes (G*ADD45A*, *IGFBP3*, and *SIAH1*). The circles indicate the nodes (genes) with the edges that are solid lines indicating the interaction scores of PPIs. The sizes of circles indicate the values of |log_2_(FC)|, while the red and blue colors indicate the upregulated and downregulated statuses, respectively.

The interaction scores of PPIs are listed in **Supplementary File S3**, which were calculated based on the STRING database (24). According to the information of the interaction scores, log_2_(FC) values, and regulation statuses, the PPI networks of extremely downregulated *HSPA8*, *HSPA2*, *HSPA1B*, and *ARRB1* and upregulated *GADD45A*, *IGFBP3*, and *SIAH1* are visualized in **Figures 5**, **6**.

In **Figure 5**, the four downregulated DE genes were connected using *HSPA2* and *HSPA8* as nodes with the PPI scores of *HSPA1B*-*HSPA2*, *HSPA2*-*HSPA8*, and *HSPA8*-*ARRB1* equal to 0.61, 0.92, and 0.64, respectively, where the connecting joint for *HSPA1B* (PPI score = 0.4) and *ARRB1* (PPI score = 0.42) was downregulated *GSK3B*. It also showed that *HSPA8* had close connections with *HSPA1B*, *HSPA2*, and *ARRB1* by both downregulated and upregulated nodes. The extremely downregulated *TUBB4A* [log_2_(FC) = -1.28, adjusted *P*-value = 2.16 × 10^-8^) and top one upregulated *PPP1R15A* [log_2_(FC) = 1.51, adjusted *P*-value = 2.16 × 10^-43^] were also connected with *HSPA8* with PPI scores of 0.57 and 0.47, respectively (**Figure 5**).

In **Figure 6**, it is shown that *GADD45A* had a strong relationship with *CCNB2* (PPI score = 0.9) in upregulated status and a moderate relationship with *ZMAT3* (PPI score = 0.46) in downregulated status. *GADD45A* was connected with *PERP* (PPI score = 0.45) that was also in a relationship with *IGFBP3* (PPI score = 0.43). *IGFBP3* was closely related to upregulated *CXCL8* (PPI score = 0.46) and downregulated *ACTB* (PPI score = 0.59); meanwhile, the downregulated *CTNNB1* was the connecting node for *IGFBP3* (PPI score = 0.54) and *SIAH1* (PPI score = 0.99). The relationship among *GADD45A*, *IGFBP3*, and *SIAH1* was built by *EP300* with PPI scores equal to 0.94, 0.92, and 0.43, respectively (**Figure 6**).

### Validated candidate differentially expressed genes by qRT-PCR experiment

**Figure 7:**
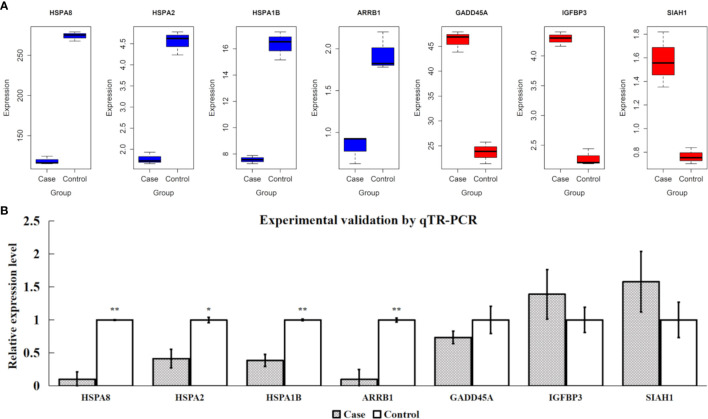
Validations for four extremely downregulated differentially expressed (DE) genes (*HSPA8*, *HSPA2*, *HSPA1B*, and *ARRB1*) and three extremely upregulated DE genes (*GADD45A*, *IGFBP3*, and *SIAH1*). **(A)** Gene expression of FPKM values, where the blue and red colors indicate the downregulated and upregulated genes, respectively. **(B)** Experimental validations by qTR-PCR. ** and * indicate *P*-value <0.01 and *P*-value <0.05, respectively, after Student’s *t*-test.

Based on the FPKM values of all samples, the expression levels of four extremely downregulated DE genes (H*SPA8*, *HSPA2*, *HSPA1B*, and *ARRB1*) and three extremely upregulated DE genes (G*ADD45A*, *IGFBP3*, and *SIAH1*) are visualized in **Figure 7A**. It is shown that the samples of downregulated DE genes had significantly higher expression levels in the control group than in the case group and *vice versa* (**Figure 7A**). After the experimental validations by qTR-PCR, we found that the four downregulated DE genes showed consistent tendencies with the transcriptomic results, and their relative expression levels were also significantly different between the two compared groups (*P*-value < 0.05). However, one of the three upregulated DE genes (*GADD45A*) showed an inconsistent tendency, and a lower expression level was observed in the case group (**Figure 7B**). In addition, the protein expression levels of HSPA8, HSPA1B and HSPA2 were investigated using western blotting technique to indicate their protein levels were significantly different between two groups (*P*-value < 0.05); meanwhile, HSPA8, HSPA1B and HSPA2 protein results were also consistent with qTR-PCR and transcriptomic results (**Supplementary Figure S2**).

## Discussion

The enzymatic role of pyruvate kinase (PK) is to catalyze the irreversible conversion of phosphoenolpyruvate (PEP) to pyruvate by the transfer of a high-energy phosphate from PEP to ADP to form ATP. In mammals, four different isoforms of PK (PKM1, PKM2, PKL, and PKR) were identified (4). All isoforms have a tetrameric form, while only PKM2 has a dimeric form besides the tetrameric form (4). As the terminal and last rate-limiting enzyme in the glycolytic pathway, PKM2 with its unique isoforms plays a crucial role in glycolytic reprogramming, which reconfigures cellular anabolic requirements and thus potentially induces metabolic transformation to alter cell identity and fate (4, 5, 27). The dimer state of PKM2 has low enzymatic activity and shifts the glycolytic flux into branching pathways, such as polyol pathway, pentose phosphate pathway, and uronic acid pathway, thus promoting anabolic function (glycogen synthesis, glycerol synthesis, nucleotide synthesis, or amino acid biosynthesis); the main purpose of the tricarboxylic acid (TCA) cycle is no longer to supply H^+^ and REDOX equivalents to the electron transport chain but to provide carbon source and REDOX equivalents for the subsequent anabolic metabolism. Consistent with this concept, the pro-proliferative role of dimeric PKM2 in proliferating cells, such as embryos or cancer cells, has been widely known (7, 28). Beyond the canonical enzymatic function, dimeric PKM2 could be translocated into the nucleus and act non-glycolysis enzyme function by regulating gene transcription (8), while PKM2 tetramer is located in the cytoplasm with a higher affinity with its substrate PEP to play the enzymatic role of PK that is related to ATP synthesis and catabolism known as PKM2 paradox in the Warburg effect (28). PKM2 interconnects glycolysis together with catabolic and anabolic processes, coordinating with the elaborate complementary metabolism of the TCA cycle intermediates.

**Figure 8:**
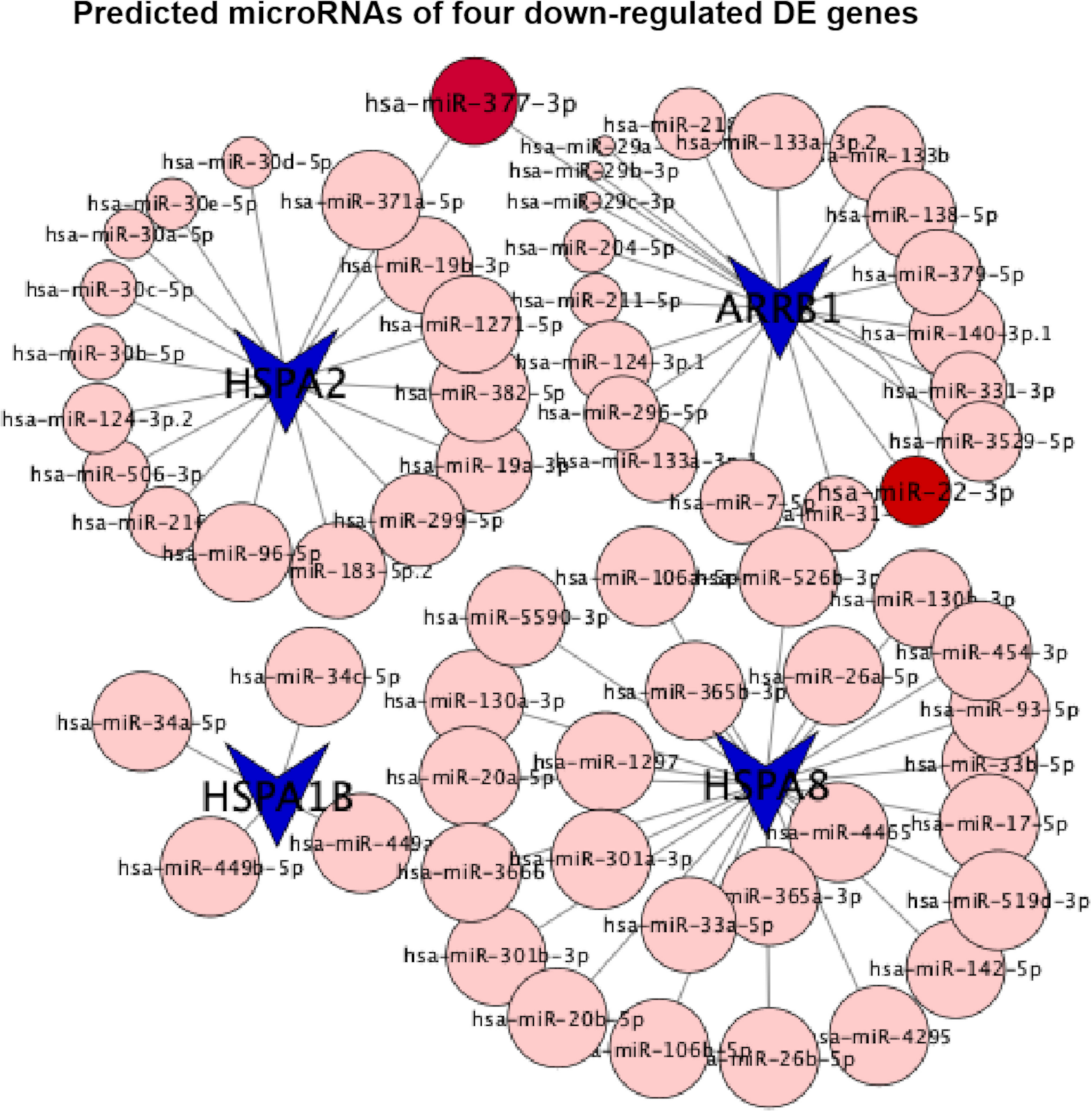
Predicted microRNAs (conserved sites) of four extremely downregulated differentially expressed (DE) genes (H*SPA8*, *HSPA2*, *HSPA1B*, and *ARRB1*). The circles indicate the context++score percentiles of the predicted microRNAs, where hsa-miR-377-3p is visualized with the score equal to 87 predicted by *HSPA2*.

The activation of glycolytic PKM2 by TEPP-46, reducing PKM2 dimerization, could prevent against fibrosis development and progression by reducing *de novo* glycine synthesis to downregulate collagen synthesis and secretion in myofibroblasts (29). Dimeric PKM2 located in the nucleus would stabilize the transcription hypoxia-inducible factor 1-alpha (HIF-1α) and directly phosphorylate the signal transducer and activator of transcription 3 (STAT3) to activate the transcription of several phosphorylated STAT3-targeted genes (8). In diabetic HK-2 cells, TEPP-46 decreased HIF-1α and phosphorylated STAT3 accumulations by decreasing the dimer PKM2 and inhibiting fibronectin, type I collagen α3, and TGF‐β1 expression, there and then preventing their downstream hypoxia and fibrosis (8). Here we intervened HK-2 cells under sustained HG exposition (7 days) with (case group) or without (control group) the addition of TEPP-46 for another 1 day and analyzed the genome-wide transcriptome data from the case and control groups. The results of our bioinformatics analyses showed the downregulation of the endocytosis pathway. Consistent with this, three of the top 10 downregulated DE genes (*HSPA8*, *HSPA2*, and *HSPA1B*) were involved in this pathway. Notably, all of them belong to the ubiquitous heat shock protein 70 (HSP70) family, and the downregulation of these three DE genes had been verified by qTR-PCR experimentsand further by western blotting experiments (**Figure 7** and **Supplementary Figure S2**). In addition, the four extremely downregulated DE genes (H*SPA8*, *HSPA2*, *HSPA1B*, and *ARRB1*) predicted 65 microRNAs in the conserved sites (**Figure 8**), with the context++score percentiles ranging from 20 to 99 (**Supplementary File S3**). Most of the microRNAs (*n* = 25) were predicted by *HSPA8*, while *HSPA1B* only predicted four microRNAs. We found that 17 and 20 microRNAs were predicted for *HSPA2* and *ARRB1*, where hsa-miR-377-3p was predicted by these two downregulated DE genes with a score equal to 87 and 47, respectively. Two binding sites of hsa-miR-22-3p were predicted by *ARRB1* in the positions of 429–436 and 4176–4182 in the 3′UTR region with a score equal to 90 and 70, respectively (**Figure 8**).

The HSP70 family (Hsp70s) acts in a large variety of cellular housekeeping functions (30). Hsp70s participates in virtually all stages of the life of proteins through the allosteric control of their client substrates, cooperating with various other cellular machineries to perform the processes of protein folding/unfolding, membrane translocation, and aggregation/disaggregation (30, 31). Hsp70s could recognize and bind a KFERQ-like pentapeptide motif in the substrate proteins, making them the key proteins required for chaperone-mediated autophagy (CMA) to deliver cargo to lysosomes. CMA degrades target soluble proteins in a selective manner and is the predominant form of autophagy in the tubular system (32, 33). Hsp70s would regulate autophagic flux through the ubiquitin-mediated proteasome system and also the autophagosome–lysosome fusion system (34). Hsp70s also take part in receptor-mediated endocytosis, impacting their internalization and subsequent delivery to endosomes and lysosomes for ligand processing and receptor degradation or recycling (30). Overall, Hsp70s play the orchestrating and pleiotropic roles in dynamic versatile networks contacting endocytosis, autophagy, and lysosomes.

*HSPA8* was the most downregulated DE gene [log2(FC) = -1.12] and also one core hub in PPI networks in this study. Located on chromosome 11q24.1, *HSPA8* encodes the human soluble constitutive 71-kDa heat shock cognate protein, also known as Hsc70 (35). *HSPA8* had been identified as the uncoating ATPase of clathrin-coated vesicles, which would disassemble the clathrin cages to modulate the clathrin-coated vesicle cycle and endocytic trafficking, acting as the essential late step in clathrin-mediated endocytosis (36). Accumulation of CMA-related HSPA8 proteins was observed in aged mutant striatum along with increased GAPDH and clustered lysosomes (32). Wen et al. (2020) reported that HSPA8 was upregulated in exosomes derived from high-glucose-treated renal tubular cells which would stimulate the proliferation and activation of fibroblasts to progress to fibrosis (37). Johnson et al. (2009) reported that HSPA8 could be co-precipitated with pyruvate dehydrogenase, isocitrate dehydrogenase, and the mitochondrial protease OMI in the isolated mitochondria of human embryonic kidney 293 cells, indicating a direct role of HSPA8 in the TCA cycle (38). *HSPA1B* and *HSPA2*, also known as HSP70-1/1B and HSP70-2/3, locate on chromosome 6p21.33 and 14q23.3, respectively; both of them encode for the classical Hsp70s proteins. *HSPA1B* was adaptively increased under salt stress in renal tubular and osmotic stress in collecting duct cells of rodents (39, 40). *HSPA2* has prominent roles in the morphological differentiation of male germ cells as well as their functional transformation during post-testicular sperm maturation (41). Zhu et al. (2021) revealed the involvement of HSPA2 signaling pathway in fibroblast pathologies in diabetic wounds through integrated bioinformatic analyses (42). To enable to fulfil their multifarious influences on cell fate, Hsp70s has similarities in structure and redundancy in function; meanwhile, they also exhibit their own structural and biochemical characteristics, exhibiting pleiotropic and context-dependent roles in cells (30, 43, 44). Hsp70s, including *HSPA8*, *HSPA2*, and *HSPA1B*, could be either tumor-promoting or tumor-suppressing depending on cell identity and context (45–47). *HSPA8* was involved in AQP2 internalization; coordinately, *HSPA1B* was likely to play a role in AQP2 trafficking to the apical plasma membrane (48). *HSPA1B* had been identified as one disease-relevant molecular target in advanced-stage Parkinson’s disease (49). Upregulated *HSPA2* exerted a neuroprotective effect through its regulation of endocytosis in a rat middle cerebral artery occlusion model (50), and inhibition of *HSPA8* would protect against spinal ischemia–reperfusion injury *via* astrocyte NF-κB/NLRP3 inflammasome pathway (51).

The endocytosis pathway controls the orchestra of myriad and specialized transport systems in PTECs; thus, it gates the internalization of extracellular substances and cell surface proteins, participates in processes including nutrient uptake, ion transport, junction formation, cellular properties, and signal transduction, and affects renal disease progression and multisystem complications (52, 53). The endocytosis pathway seems to function as a double-edged sword in PTECs: on one hand, endocytosis plays a vital role in the efficient reabsorption of excess ultra-filtered ions and solutes; on the other hand, it leads to the susceptibility of PTECs to excess nutrients and environmental stress. Under diabetic stress, the sustained activation of endocytosis would be uncoordinated with impaired energy metabolism and excessive oxidative stress, which further promotes and sustains dominos of pathological injuries. Excessive mobilization of membrane phospholipids associated with enhanced endocytosis would overwhelm the degradation capacity of the autophagy–lysosome system in PTECs (54).

The endocytic cycle, together with HSP70s, integrates pleiotropic and dynamic cellular roles in response to the internal and external environment. In *Drosophila melanogaster*, clathrin-uncoating ATPase Hsc70-4 could interact genetically with the activated mechanistic target of rapamycin (mTOR) signaling, leading to an increase in bulk endocytosis and a decrease in the targeted endocytic degradation of excess nutrient importers in cells and together increasing the intracellular nutrient burden (55). mTORC1 regulated endocytosis and nutrient transport in rodent proximal tubular cells and played a central role in the pathophysiology of DKD (9, 56). In diabetic proximal tubular cells, the increased mTORC1 activity and relevance of enhanced amino acid transport and probably decreased branched-chain amino acid degradation accelerated fibrogenesis (9). Recently, the activation of mTORC1 had been tied to intracellular glycolysis state. Orozco et al. (2020) reported that mTORC1 sensed metabolites downstream of aldolase and upstream of the GAPDH-catalyzed steps of glycolysis and pinpointed dihydroxyacetone phosphate (DHAP) as the key and sufficient metabolite to activate mTORC1 even in the absence of glucose (57). Thus, here we deduced that decreased mTORC1 activity by reducing the accumulation of glycolytic intermediate DHAP responding to TEPP-46 might be partly tied to the downregulation of Hsp70s (*HSPA8*, *HSPA2*, and *HSPA1B*) network and certain endocytosis pathways in hyperglycemic HK-2 cells, which would circumvent metabolic stress and remodel homeostasis between enhanced intracellular transport and impaired metabolic capacity to the natural potency.

The coordination of cellular metabolic remodeling and endocytic pathways represents an important feature of tubular cellular plasticity and adaptive homeostasis (52). Our bioinformatics analyses showed that the epidermal growth factor receptor (*EGFR*) was simultaneously involved in four downregulated pathways (adherens junction, regulation of actin cytoskeleton, endocytosis, and EGFR tyrosine kinase inhibitor resistance). EGFR trafficking is a key regulator in cell proliferation, differentiation, division, and survival, and EGFR internalization occurs *via* endocytosis by its recruitment into clathrin-coated pits (58, 59). Numerous studies had demonstrated that transient activation of the EGFR pathway was required for promoting kidney recovery from acute injury, whereas sustained activation of EGFR in PTECs under diabetogenic stimuli was sufficient to induce epithelial–mesenchymal transition and cause spontaneous and progressive renal tubulointerstitial fibrosis (10, 60). Here we proposed that PKM2 activator TEPP-46 could alter the intracellular trafficking of EGFR signaling cascade partly through its impact on endocytosis, in which the downregulated Hsp70s (*HSPA8, HSPA2*, and *HSPA1B*) were involved, presenting a new perspective on remodeling cellular mechanics and dynamics through metabolic phenotypes. However, functional research is still required for the candidate genes in this study to validate their molecular mechanism of *in vitro* reprogramming under hyperglycemic stress that orchestrates HK-2 cell functions.

## Conclusions

Our study treated hyperglycemic HK-2 cells with PKM2 activator TEPP-46 and conducted genome-wide transcriptome analysis for the case/control group. Finally, we identified 2,902 DE genes including 87 and 56 extremely downregulated and upregulated DE genes (adjusted *P*-value ≤ 0.05 and |log_2_(FC)| ≥ 1). In addition, 2,509 DE genes among them (86.46%) were co-expressed in the key module. Four extremely downregulated DE genes (*HSPA8*, *HSPA2*, *HSPA1B*, and *ARRB1*) and three extremely upregulated DE genes (*GADD45A*, *IGFBP3*, and *SIAH1*) enriched in the downregulated endocytosis (hsa04144) and upregulated p53 signaling pathway (hsa04115), respectively, were validated by the qTR-PCR experiments, where the relative expression levels of four extremely downregulated DE genes were significantly different (*P*-value < 0.05) between the case and control groups. Here we shed light that Hsp70s (*HSPA8*, *HSPA2*, and *HSPA1B*) are the key transcriptomic biomarkers responding to the PKM2 activator TEPP-46. Our results together highlight a possibility that PKM2 tetramerization induced by TEPP-46 in hyperglycemic HK-2 cells would reshape the interplay among endocytic trafficking, dynamics of protein folding–unfolding, and autophagy–lysosome system through the versatile networks of Hsp70s, rewiring the crosstalk between EGFR signal transduction circuits and metabolic stress to promote resilience.

## Data availability statement

The raw RNA sequencing data were deposited in the Gene Expression Omnibus (GEO) of National Center for Biotechnology Information (NCBI) with the accession number GSE205674 at https://www.ncbi.nlm.nih.gov/geo/query/acc.cgi?acc=GSE205674.

## Author contributions

XW and ZW conceived and designed the experiments. JY, XL, and ZW performed the experiments. ZW, JY, DH, and XW analyzed the data and wrote the manuscript. ZW, DH, XL, and XW improved the manuscript. ZW and JY contributed equally to this manuscript. All authors contributed to the article and approved the submitted version.

## Funding

This study was supported by the Union Program of the Key Scientific and Technological Project of Henan Province (grant no. 2018020107) and the National Natural Science Foundation of China (grant nos. 82100896).

## Conflict of interest

XW was employed by the company Konge Larsen ApS. DH was employed by Shijiazhuang Zhongnongtongchuang (ZNTC) Biotechnology Co., Ltd.

The remaining authors declare that the research was conducted in the absence of any commercial or financial relationships that could be construed as a potential conflict of interest.

## Publisher’s note

All claims expressed in this article are solely those of the authors and do not necessarily represent those of their affiliated organizations, or those of the publisher, the editors and the reviewers. Any product that may be evaluated in this article, or claim that may be made by its manufacturer, is not guaranteed or endorsed by the publisher.

## Supplementary material

Supplementary Table S1 The primers used in the qRT-PCR validation.

Supplementary Figure S1 R^2^ of the free-scale topology and mean connectivity with soft threshold (power) for 21,067 genes.

Supplementary Figure S2 The protein expression levels of HSPA8, HSPA1B and HSPA2. ** and * indicate *P*-value < 0.01 and *P*-value < 0.05, respectively, after Student’s *t*-test.

Supplementary File S1 Fragments per kilobase million mapped reads for each gene (*n* = 29,483) along six different samples.

Supplementary File S2 All 2,902 differentially expressed (DE) genes including 143 extremely DE genes with chromosome positions, gene names, gene descriptions, read counts for each gene along different samples, log_2_(FC) values, *P*-values, adjusted *P*-values, and regulation status along six different samples.

Supplementary File S3 Interaction scores of protein–protein interactions and the context++score percentiles of the predicted microRNAs for four extremely downregulated differentially expressed genes (*i*.*e*., *HSPA8*, *HSPA2*, *HSPA1B*, and *ARRB1*).
