# Supplementary material for: Transcriptomic signatures responding to PKM2 activator TEPP-46 in the hyperglycemic human renal proximal epithelial tubular cells
Source: Front Endocrinol (Lausanne). 2022 Aug 31;13:965379. doi: 10.3389/fendo.2022.965379 (PMC9471676; doi:10.3389/fendo.2022.965379)
Supplement: Supplementary Table S1 — The primers used in the qRT-PCR validation. [file Table_1.docx]

Supplementary Table S1. The primers used in the qRT-PCR validation.

| Gene name | Forward primer | Reverse primer | Length |
| --- | --- | --- | --- |
| *GAPDH* | ACCCAGAACCATGCAAATCACA | ACCCAGAACCATGCAAATCACA | 168 |
| *HSPA8* | CTGTGGACAAGAGTACGGGAAA | CCTTGTCCCTCTGCTTCTCATCT | 138 |
| *HSPA2* | GACGTGTCCATCCTGACCATC | CGAAAGAGGTCGGCATTGAG | 317 |
| *HSPA1B* | GAGCAGGTGTGTAACCCCAT | CAGCAAAGTCCTTGAGTCCCA | 192 |
| *ARRB1* | CGAGCACGCTTACCCTTTCA | TCTTCTCCTCCAAATTCTCCGC | 146 |
| *GADD45A* | AGAAGACCGAAAGCGACCCC | GATGTTGATGTCGTTCTCGCAG | 134 |
| *IGFBP3* | CTCAGAGCACAGATACCCAGAAC | AGGCTGCCCATACTTATCCAC | 239 |
| *SIAH1* | GGCTACTCCACCTTCTCTGTACTCC | GGATGCAGTTGTGCCAGTCAG | 175 |


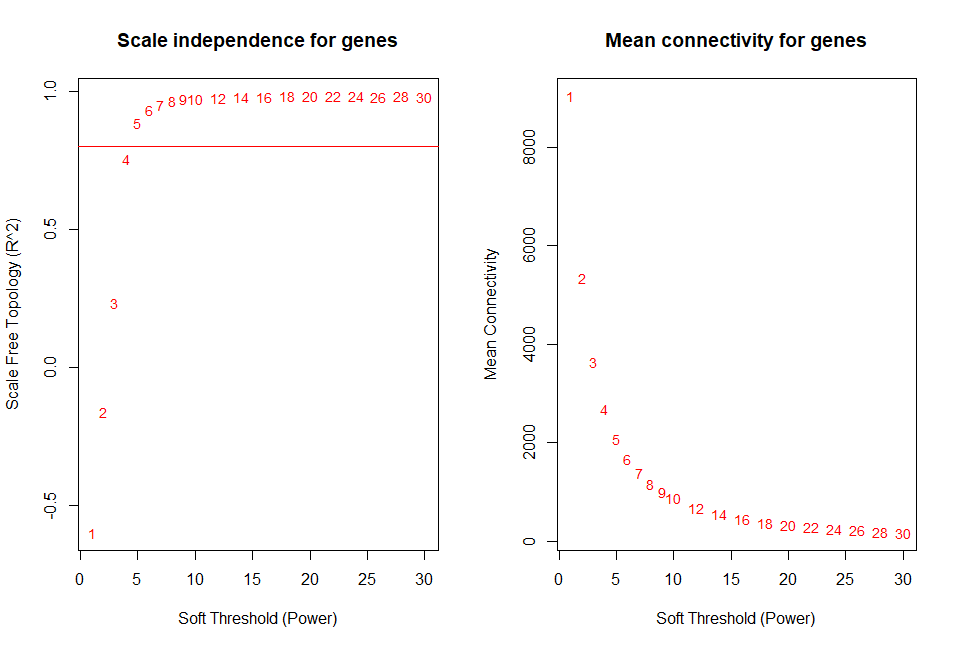
Supplementary Figure S1. R^2^ of the free-scale topology and mean connectivity with soft threshold (power) for 21,067 genes.


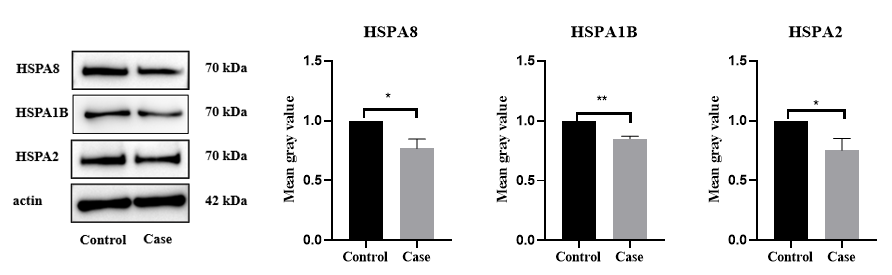


Supplementary Figure S2. The protein expression levels of HSPA8, HSPA1B and HSPA2. ** and * indicate *P*-value < 0.01 and *P*-value < 0.05, respectively, after Student’s *t*-test.
